# A Wolf in Sheep’s Clothing: Pseudohypobicarbonatemia in a Patient With Multiple Myeloma

**DOI:** 10.7759/cureus.80782

**Published:** 2025-03-18

**Authors:** Ziad Alahmadi, Zain Haq, Ami Patel, Joshua D King

**Affiliations:** 1 Internal Medicine, Umm Al-Qura University, Makkah, SAU; 2 Nephrology, Samaritan Health Services, Corvallis, USA; 3 Nephrology, University of Maryland Medical Center, Baltimore, USA

**Keywords:** acid-base disorders, errors in the laboratory processes, high anion gap metabolic acidosis, multiple myeloma, serum bicarbonate

## Abstract

There are multiple methods to measure serum bicarbonate level which is crucial for diagnosis and management of acid-base disturbances. The most common method is directly measuring the total serum carbon dioxide (CO_2_) concentration in a basic metabolic panel. Another method is by indirect calculation of the bicarbonate concentration via the Henderson-Hasselbalch equation using the measured pH and partial pressure of CO_2_ (pCO_2_) in plasma. Multiple confounders can cause discrepancies in the measured bicarbonate in each method, such as increased concentration of serum lipids or proteins, that can alter measurement assays, leading to spuriously low serum bicarbonate values. In our case, we present a patient with falsely low reported bicarbonate levels in the setting of increased paraproteins due to multiple myeloma.

## Introduction

A low bicarbonate level in the basic metabolic panel (BMP) can be used usually as an initial value to screen for acid-base abnormalities. While there are multiple methods to measure serum bicarbonate level which is crucial for the diagnosis and management of acid-base disturbances, the most common method is by directly measuring the total serum carbon dioxide (CO_2_) concentration in a BMP via measuring total CO_2_ in the blood, as approximately 95% of total CO_2_ in the blood is in HCO_3_^-^ form [1,2]. To measure the total CO_2_, there are multiple chemistry analyzers that can use either enzymatic/photometric or electrode-based assays for measurement [1,2]. In our institution, we use Vitros® 350 Chemistry Analyzer (Ortho Clinical Diagnostics, Raritan, New Jersey, USA), which measures bicarbonate via carboxylation of phosphoenolpyruvate with phosphoenolpyruvate carboxylase (PEPC) to form oxaloacetate and inorganic phosphate. Oxaloacetate then converts to malate via malate dehydrogenase, which will produce nicotinamide adenine dinucleotide (NAD) from reduced nicotinamide adenine dinucleotide (NADH) reduction. The concentration of CO_2_ is then identified by measuring the absorbance of unreacted NADH by spectrophotometry as described in the manufacturer guide [3]. An alternative method to figure the HCO3 value is by indirect calculation of the bicarbonate concentration via the Henderson-Hasselbalch equation using the measured pH and partial pressure of CO_2_ (pCO_2_) in plasma.

Monoclonal gammopathy has been reported to cause alteration in anion gap as it can act as a cation or anion based on the paraprotein charge [4,5]. In the literature, elevated IgG has been reported to be cations causing lower anion gap than expected while elevated IgA or free light chains will cause elevated anion gap as it will act as anions [5]. In our case, we present a patient with a falsely low reported bicarbonate level with a high anion gap in the setting of increased paraproteins due to multiple myeloma.

## Case presentation

A 47-year-old male patient with a history of IgA lambda chain multiple myeloma, cast nephropathy with chronic kidney disease (CKD), and hypertension presented with hypoxia, requiring high-flow oxygen that was attributed to pneumonia and volume overload from significant hypertension. Although he had clinical improvement of his hypoxia with antibiotics, intravenous (IV) diuretics, and hypertension treatment, he was noted to have a significantly low total CO_2_ level on the initial BMP ranging 8-11 mmol/L (normal: 21-30 mmol/L) with anion gap of 20-26 mmol/L. He was presumed to have a high anion gap metabolic acidosis from his acute CKD and was at first treated with an IV bicarbonate infusion with no significant change in reported CO_2_ value. He had normal lactate and normal blood sugar, and no apparent contributing factor was detected in history or physical examination that would explain this acid-base disturbance.

A venous blood gas (VBG) was performed, which showed a pH of 7.36, pCO_2_ of 42 mmHg, and calculated bicarbonate of 22 mmol/L; a simultaneous metabolic panel showed CO_2_ of 10 mmol/L and anion gap of 23. Lambda light chain was significantly elevated at 1788.97 mg/L and IgA level of 3804 mg/dL. The patient was started on chemotherapy (carfilzomib/dexamethasone/cyclophosphamide regimen), which led to the reduction of both lambda light chains and IgA levels to 812.98 mg/L and 2276 mg/dL, respectively, within one week of initiating treatment, which was reflective to improved accuracy of bicarbonate levels in BMP compared to VBG with the reduction of paraproteins (Table 1).

**Table 1:** Impact of paraprotein reduction on anion gap and HCO3 measurement HCO_3_: bicarbonate; BMP: basic metabolic panel; VBG: venous blood gas; CO_2_: carbon dioxide; IgA: immunoglobulin A; MM: multiple myeloma

Test	Day 1 of MM treatment	Day 7 of MM treatment
HCO_3_ from BMP	10 mmol/L	19 mmol/L
Anion gap from BMP	23	13
pH from VBG	7.35	7.41
CO_2_ from VBG	42 mmHg	37 mmHg
HCO_3_ from VBG	22 mmol/L	23 mmol/L
Lambda light chain	1788.97 mg/L	812.98 mg/L
IgA level	3804 mg/dL	2276 mg/dL

## Discussion

The difference in this patient's bicarbonate level between VBG and chemistry panel was explained by increased paraproteins, which have resulted in artifactual errors in PEPC-based laboratory analysis of serum bicarbonate. Manufacturer guide mentions total protein above 11 g/dL can impact the accuracy of the enzymatic assay and in our case, total protein was 12.8 g/dL. Concomitantly, IgA contributed as an unmeasured anion to explain the high anion gap. Discrepancies between serum and whole blood bicarbonate have also been reported in the literature due to various reasons including elevated triglycerides [6], increased paraproteins interfering with enzymatic assay as in our case [7], and tourniquet-induced venous stasis causing CO_2_ accumulation [8]. In our case, the patient’s anion gap and accuracy of serum bicarbonate level measurement improved as their lambda light chain levels subsequently decreased. Additionally, IgA can act as an unmeasured anion contributing to a high anion gap, which was evident in our case, this has been a documented phenomenon in previous studies [9,10]. Our patient anion gap has markedly decreased with the reduction of IgA level, which supports the literature findings.

Laboratory analytical artifacts leading to discrepancies between direct and indirect measurements of HCO_3_ have been rarely reported but can lead to unwarranted interventions. The appreciation of the patient, collection, and analysis of specific factors is crucial for accurate interpretation to avoid misdiagnosis or overtreatment based on spurious laboratory findings [11].

## Conclusions

Interpretation of serum CO_2_ is usually accurate; rarely, there are factors that can cause false readings including elevated paraproteins and triglycerides. An arterial or VBG bicarbonate value can guide you to detect pseudohypobicarbonatemia in the appropriate clinical setting.
